# Prediction of COVID-19 disease progression by multiparametric analysis of circulating extracellular vesicles with flow cytometry

**DOI:** 10.1128/jvi.01189-25

**Published:** 2025-09-23

**Authors:** Evelyn Hammer, Charlotte Flynn, Johannes Rößler, Johanna Erder, Rudolf Napieralski, Lisa Fricke, Birgit Campbell, Martin Feuerherd, Felix Esslinger, Albrecht von Brunn, Timm Weber, Siobhan King, Sisareuth Tan, Alain R. Brisson, Ulrike Protzer, Gabriele Schricker, Kathrin Gärtner, Gregor Ebert, Allessandra Moretti, Florian Klein, Kevin Knoops, Ron Heeren, Wolfgang Hammerschmidt, Reinhard Zeidler, Olaf Wilhelm, Percy A. Knolle, Bastian Höchst

**Affiliations:** 1 Institute of Molecular Immunology, University Hospital München rechts der Isar, School of Medicine and Health, Technical University Munich (TUM) 155892, Munich, Germany; 2 therawis diagnostics GmbH, Munich, Germany; 3 Research Unit Gene Vectors, Helmholtz Zentrum München, Munich, Germany; 4 TUM School of Medicine and Health, Department of Clinical Medicine – Clinical Department for Internal Medicine II, University Medical Center, Technical University of Munich 9184, Munich, Germany; 5 Medical Department – Molecular Cardiology, Technische Universität München 9184, Munich, Germany; 6 Institute of Virology, Technische Universität München/Helmholtz Zentrum München, Munich, Germany; 7 Max von Pettenkofer-Institut, Ludwig-Maximilians-Universität München 9183, Munich, Germany; 8 Laboratory of Experimental Immunology, Institute of Virology, Faculty of Medicine and University Hospital Cologne, University of Cologne 14309, Cologne, Germany; 9 Oxford Nanoimaging ONI 551994 , Oxford, United Kingdom; 10 UMR-5248 CBMN CNRS-University of Bordeaux-IPB, Allée Geoffroy Saint-Hilaire, Pessac, France; 11 German Centre for Infection Research (DZIF), Munich, Germany; 12 Institute for Advanced Science, Technische Universität München 9184, Munich, Germany; 13 Eximmium Biotechnologies GmbH, Munich, Germany; 14 Centre for Infection Research (DZIF), Partner Site Bonn-Cologne, Cologne, Germany; 15 The Microscopy CORE Lab, Maastricht Multimodal Molecular Imaging Institute, Maastricht University 5211, Maastricht, the Netherlands; 16 Institute of Molecular Immunology, School of Life Sciences, Technische Universität München 9184, Munich, Germany; 17 German Center for Infection Research Munich Site 655317 , Munich, Germany; St. Jude Children's Research Hospital, Memphis, Tennessee, USA

**Keywords:** SARS-CoV2, COVID-19, extracellular vesicles, virosome, flow cytometry, progress prediction

## Abstract

Extracellular vesicles (EVs) are released from all cells of the body. They are considered to mirror the state of the cells from which they are released and circulate in the blood, suggesting a possible use of EV analysis for diagnostic purposes. Here, we report that the analysis of single EVs by flow cytometry can detect infection of cells with severe acute respiratory syndrome coronavirus 2 (SARS-CoV-2) by identifying expression of the SARS-CoV-2 spike (S) protein on the surface of EVs and the cellular origin of EVs by detecting cell-type-specific markers such as troponin (cTNT1) for cardiomyocytes. In coronavirus-associated disease 19 (COVID-19) patients, we detected a direct correlation of the frequencies of circulating S-expressing EVs, but not of cTNT/S-co-expressing EVs, with the subsequent development of a severe disease course. Detection of circulating S-expressing EVs indicates widespread SARS-CoV-2 infection in the body, which may contribute to the immune pathogenesis that triggers tissue and organ damage in COVID-19. Our findings suggest that detecting circulating viral antigen-expressing EVs may provide crucial predictive information on infection-associated disease courses in situations of a future viral pandemic.

**IMPORTANCE:**

The ability to predict which patients infected with the SARS-CoV-2 virus will develop severe disease remains a significant clinical challenge. The present study demonstrates that EVs in the peripheral blood, carrying the SARS-CoV-2 spike protein, can be detected by flow cytometry and serve as early biomarkers of disease progression. In contradistinction to PCR or serology, this method provides insight into systemic viral spread and potential organ involvement. The early identification of spike-positive EVs at the time of hospital admission has the potential to facilitate the timely identification of high-risk patients, thereby enhancing the efficacy of triage and subsequent care. This approach may also be of value in terms of facilitating a more rapid and precise response to future virus pandemics.

## INTRODUCTION

Infection with severe acute respiratory syndrome coronavirus 2 (SARS-CoV-2) can lead to coronavirus-associated disease 19 (COVID-19), which preferentially affects the upper respiratory tract and lungs as primary sites of infection (1, 2). The clinical manifestation of COVID-19 exhibits a broad spectrum, ranging from asymptomatic cases or mild respiratory symptoms to critical illness characterized by acute respiratory distress syndrome (ARDS) and multi-organ failure in some patients. In severe cases, SARS-CoV-2 infection may precipitate a hyperinflammatory response, commonly referred to as cytokine release syndrome (CRS), which contributes to widespread tissue injury. Concurrently, dysregulation of the coagulation cascade can lead to immunothrombosis and the formation of microvascular thrombi, causing organ dysfunction across multiple organ systems (3). Overall, the number of deaths caused by COVID-19 amounts to more than 14 million worldwide (4).

SARS-CoV-2 primarily infects epithelial cells of the upper airways and the lungs (5). However, because of the widespread expression on target cells of its receptors, angiotensin-converting enzyme 2 (ACE2) and transmembrane protease serine 2 (TMPRSS2), mediating cellular infection (6), SARS-CoV-2 can also infect cells in other organs beyond the lungs. These include cells in the cardiovascular system (cardiomyocytes and endothelial cells), cells in the nervous system (neurons, astrocytes), cells in the gastrointestinal tract (intestinal epithelial cells, hepatocytes, cholangiocytes, pancreatic cells), the kidney (proximal tubular epithelial cells), and cells of the reproductive tract (7). Infection of cells in these organs may contribute to severe disease courses, such as SARS-CoV-2 infection of the heart, or may be involved in persistent infection, as reported in intestinal epithelial cells in prolonged infection (8). Of note, individuals with pre-existing cardiovascular conditions are at an elevated risk of developing severe manifestations of COVID-19 (9, 10), as it has been shown that SARS-CoV-2 infection of cardiomyocytes promotes myocardial injury and triggers cardiac arrhythmias (11). Involvement of the cardiovascular system in COVID-19 patients, determined by increased levels of cardiac troponin T (cTNT) as a sign of cardiac ischemia and injury, is associated with elevated morbidity and mortality (10, 12–14). However, it is impossible to detect SARS-CoV-2 infection of cardiomyocytes in patients unless a biopsy is taken during an invasive procedure and viral RNA is detected within the tissue.

Extracellular vesicles (EVs) are released from all cells of the body and can contain molecular information from their parental cells (15). EVs circulate in the blood (16) and bear the potential to be used as sentinels for the cells from which they are secreted. Here, we present a flow-cytometry-based detection of EVs from cardiomyocytes infected with SARS-CoV-2. This flow cytometry-based analysis allowed for the detection of EVs expressing troponin and the spike antigen of SARS-CoV-2, both from *in vitro* SARS-CoV-2-infected human cardiomyocytes and in the plasma of COVID-19 patients. The number of circulating SARS-CoV-2 spike antigen-bearing EVs directly correlated with a severe COVID-19 disease course in patients, pointing toward a potential use of circulating EVs to detect viral infection of organs that warrants further investigation.

## RESULTS

### Detection of SARS-CoV-2-spike (S) and cTnT1 expressing EVs

**Fig 1:**
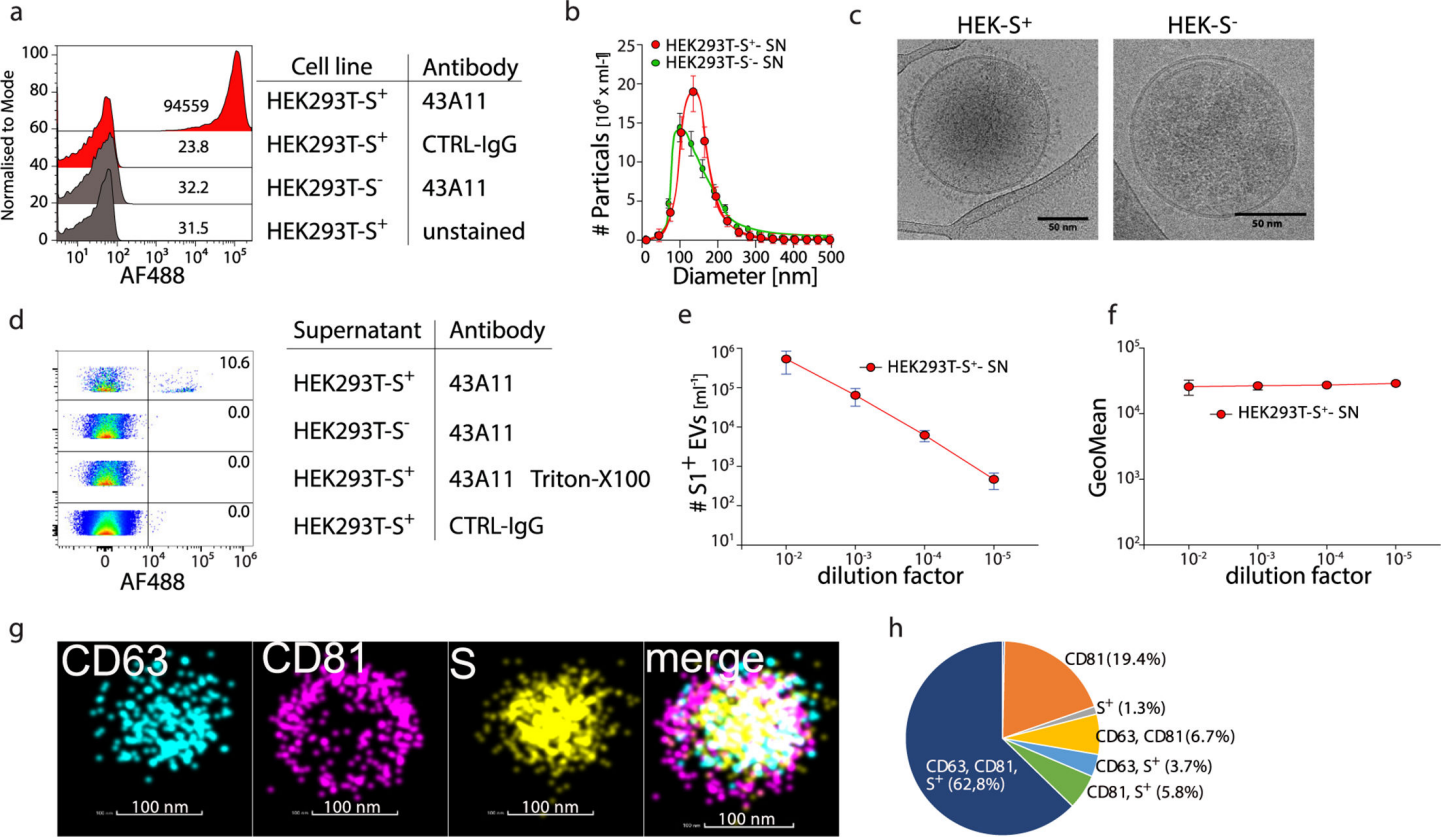
Detection of S^+^ EVs in cell culture supernatant of SARS-CoV-2 Spike-S transduced HEK 293T cells. SARS-CoV-2 S gene-transfected HEK293T cells were cultured in serum-free media for 12 h. The supernatant of HEK293T cells was centrifuged (10,000 × *g*) to remove remaining cells or fragments. For EM and dSTorm imaging, EVs were pelleted by ultracentrifugation (10^5^ × *g*) and resuspended in 10 µL of PBS. (a) Representative flow analysis of HEK293T-S^+/−^ cells stained with the S-specific clone 43A11, IgG-CTRL or were left unstained. (b) Size distribution and quantification of EVs were analyzed using nanoparticle tracking analysis (NTA). (c) Representative EM images of EVs isolated from HEK293T S^+^ and S^−^ cells (b). (d) Supernatant of HEK293T-S^+^/^−^ cells was stained with the anti-S antibody 43A11, Triton X-100, and isotype controls were included. (e and f) Serial dilution of cell culture supernatants was analyzed for count and fluorescence intensity, and the results were plotted against the dilution factor. (g and h) Representative dSTORM images of a single EV enriched from the cell culture supernatant of HEK293T S^+^ cells labeled with anti-CD63-AF568, anti-CD81-CF488, and anti-S-AF647.

We started by identifying EVs using flow cytometry and analyzing them at the level of single EVs for expression of the SARS-CoV-2 spike antigen (S) as an indicator of ongoing viral infection. For this, we transduced HEK293T cells with the *S(B.1*) gene of SARS-CoV-2, which resulted in S protein expression (Fig. 1a) and secretion of S-expressing virus-like particles, as previously described (17, 18). Nanoparticle tracking analysis (NTA) revealed a similar average size of 130 nm for EVs found in the cell culture supernatant of S-transduced and non-transduced HEK293T cells (Fig. 1b; Fig. S1a). Enrichment of EVs by ultracentrifugation (UC) from the cell culture supernatant of S-transduced HEK293T cells and subsequent Western blot analysis demonstrated S expression (Fig. S1b). Ultrastructural imaging by electron microscopy of EVs released from the S-transduced HEK293T cells into the cell culture supernatant showed round spheres enclosed by a double membrane, a characteristic feature of EVs, and the typical corona-like S antigen features on EVs from S-transduced HEK293T cells (Fig. 1c).

To analyze EVs by flow cytometry, we employed the signal from the side scatter (SSC-H) photomultiplier as a trigger for the analysis. Beyond the electronic noise signal derived from photomultipliers associated with this highly sensitive analysis, we detected S-expressing EVs, without prior enrichment by UC, in the supernatant of S-transduced HEK293T cells but not untransduced cells using a fluorochrome-labeled anti-S antibody (Fig. 1d). Side-by-side comparison of flow cytometric analysis of EVs directly from cell culture supernatant or after enrichment through UC or size exclusion chromatography (SEC) demonstrated that enrichment prior to flow cytometric analysis is not required and that direct flow cytometric analysis is even more sensitive in detecting EVs contained in cell culture supernatant (Fig. S1c and d). Serial dilution of the supernatant led to a reduction in the number of EVs detected by flow cytometry, whereas anti-S fluorescence intensity remained unchanged (Fig. 1e and f), as expected for a specific and sensitive analysis. Taken together, this demonstrates the feasibility of detecting S-expressing EVs released from S-transduced HEK293T cells by flow cytometry. To identify S^+^ events in the flow cytometric analysis as EVs, we performed a multiparameter analysis and costained for characteristic EV markers, such as CD9, CD63, and CD81, and analyzed single EVs by high-resolution dSTORM imaging. dSTORM imaging revealed colocalization of S, CD63, and CD81 on the same EV obtained from the supernatant of S-transduced HEK293T cells (Fig. 1g). Quantifying dSTORM high-resolution images of EVs from S-transduced HEK293T cells showed that approximately 75% of the vesicles co-expressed S in combination with CD63 and/or CD81 (Fig. 1h). These results revealed that a multiparametric analysis of EVs by flow cytometry is possible and allowed us to generate quantitative data on the number of EVs with a particular surface phenotype.

**Fig 2:**
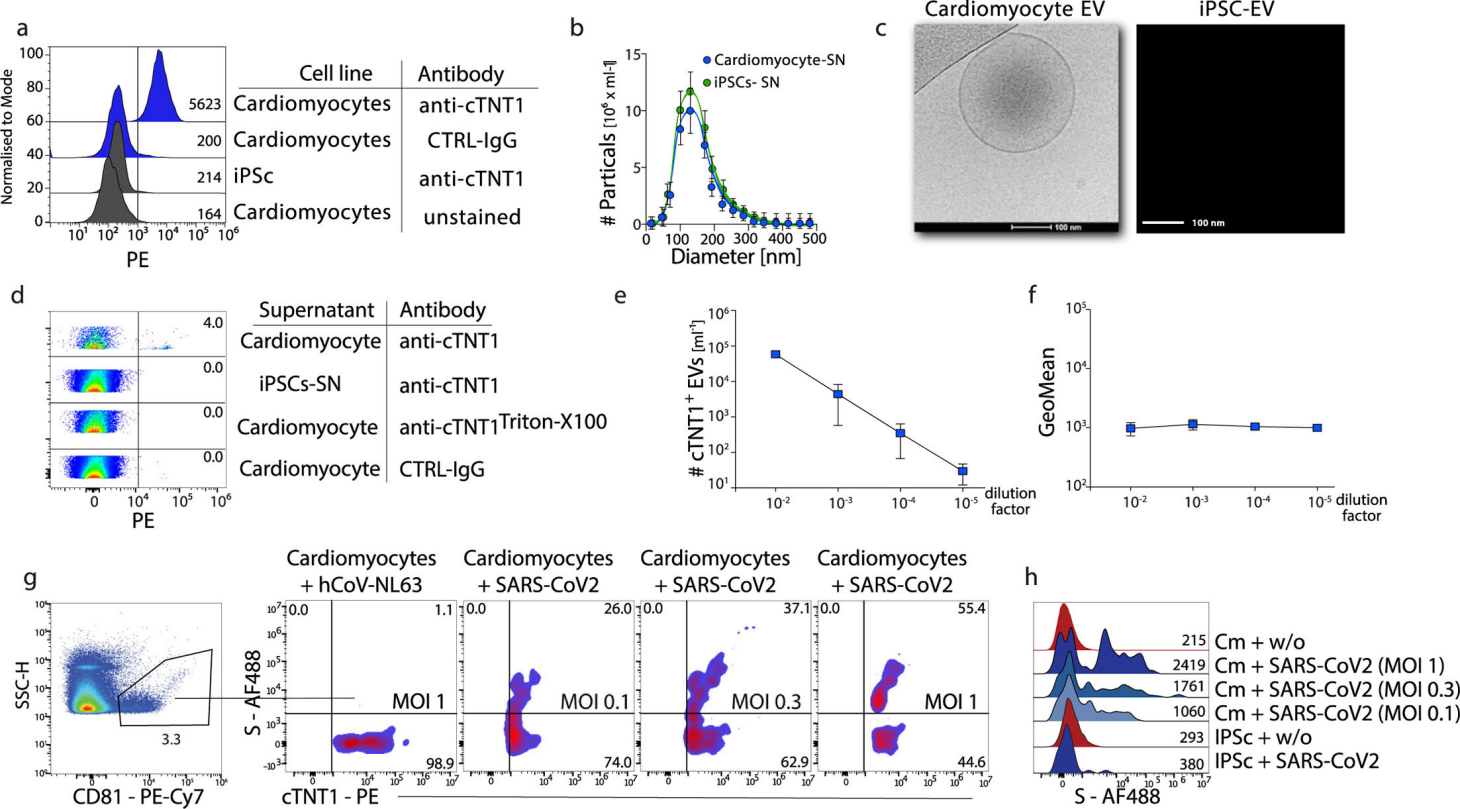
Detection of cTNT^+^ EVs in cell culture supernatant of human iPSC-derived cardiomyocytes. (a) Representative flow analysis of induced cardiomyocytes or iPSCs stained with anti-cTNT1, IgG-CTRL, or left unstained. (b) Size distribution and quantification of EVs were analyzed using nanoparticle tracking analysis (NTA). (c) Representative EM images of EVs isolated from induced cardiomyocytes or iPSCs. (d) Supernatant of cardiomyocytes was stained anti-cTNT1, Triton X-100, and Isotype controls were included. (e and f) Serial dilution of stained supernatant was analyzed for count and fluorescence intensity, and the results were plotted against the dilution factor. (g and h) Infection of cardiomyocytes with SARS-CoV-2. A confluent layer of iPSC-induced, beating cardiomyocytes that were either uninfected, infected with SARS-CoV-2 (B.1.1.7), or with hCoV-NL63 (MOI of 1). The cells were washed after 2 h, and the supernatant was collected after 12 h, followed by staining with anti-cTNT1 and anti-Spike-S antibodies, pre-gating on anti-CD81. Representative results from *n* = 3 are shown.

We proceeded to analyze cell-derived EVs for expressing an organ-specific marker, such as cTNT, released by cardiomyocytes. We used induced pluripotent stem (iPS) cells (iPSC) differentiated into functionally active human cardiomyocytes (19, 20). iPS-derived cardiomyocytes expressed the cardiomyocyte-specific marker troponin (cTNT1) (Fig. 2a). NTA revealed a similar average size of 130 nm for EVs from human cardiomyocytes as for HEK293T cells (Fig. 2b). Ultrastructural imaging of EVs released from iPSC-derived cardiomyocytes into cell culture supernatant revealed an EV characteristic double membrane feature (Fig. 2c). By flow cytometry, we detected cTNT1 expression on EVs in the cell culture supernatant of iPSC-derived cardiomyocytes using a fluorochrome-labeled anti-cTNT1 antibody (Fig. 2d), a decreased number of cTNT1^+^ EVs after serial dilution of the supernatant, and no change in anti-cTNT1 antibody fluorescence (Fig. 2e and f), altogether demonstrating the capacity to detect a cell type-specific marker on EVs by flow cytometry.

Next, we infected iPSC-derived cardiomyocytes with SARS-CoV-2 to evaluate whether S expression on cell-derived EVs can be detected by flow cytometry. We verified infection of human iPSC-derived cardiomyocytes using a recombinant SARS-CoV-2 expressing GFP (21) (Fig. S2a). Infection of iPSC-derived cardiomyocytes with SARS-CoV-2 (B.1.1.7) or the seasonal coronavirus hCoV-NL63 was confirmed by detection of viral RNA by PCR from cell lysates (Fig. S2b). Flow cytometric analysis showed that only EVs released from SARS-CoV-2-infected, but not hCoV-NL63-infected or non-infected, iPSC-derived cardiomyocytes expressed S (Fig. S2c). Importantly, S expression was identified by flow cytometry on cTNT1^+^ EVs released from SARS-CoV-2-infected cardiomyocytes, and increased frequencies of S^+^cTNT1^+^ EVs were detected with increasing numbers of SARS-CoV-2 used for infection of cardiomyocytes (Fig. 2g and h). Taken together, these results provide evidence that multiparametric analysis of individual EVs by flow cytometry allows for the simultaneous detection of cell-type-specific markers and a marker for ongoing SARS-CoV-2 infection in that cell.

### Identification of S^+^ and cTNT1^+^ EVs in the blood of COVID-19 patients

To demonstrate the feasibility of measuring circulating EVs in the blood, we first investigated plasma from healthy individuals. We analyzed EVs from plasma by flow cytometry for expression of canonical EV markers and found that they expressed CD9, CD63, CD81, and HLA-ABC (Fig. S3a). Based on these initial observations, we next investigated the potential presence of S proteins on the surface of circulating EVs from SARS-CoV-2-infected patients. We performed a clinical study and collected plasma samples from COVID-19 patients admitted to the emergency department of the TUM University hospital between 2 February 2021 and 5 June 2021. Patients did not report prior COVID-19 vaccination and did not receive treatment with anti-SARS-CoV-2 antibodies before or at the time of sampling. A total of 25 patients were included in this analysis. In 19 COVID-19 patients, SARS-CoV-2 infection was detected with the B.1.1.7 variant of concern, 2 patients with the original strain of SARS-CoV-2, and 1 patient with the B.1.351 variant of concern. In contrast, in three patients, no information on the variant of concern could be obtained (details listed in the Table S1).

**Fig 3:**
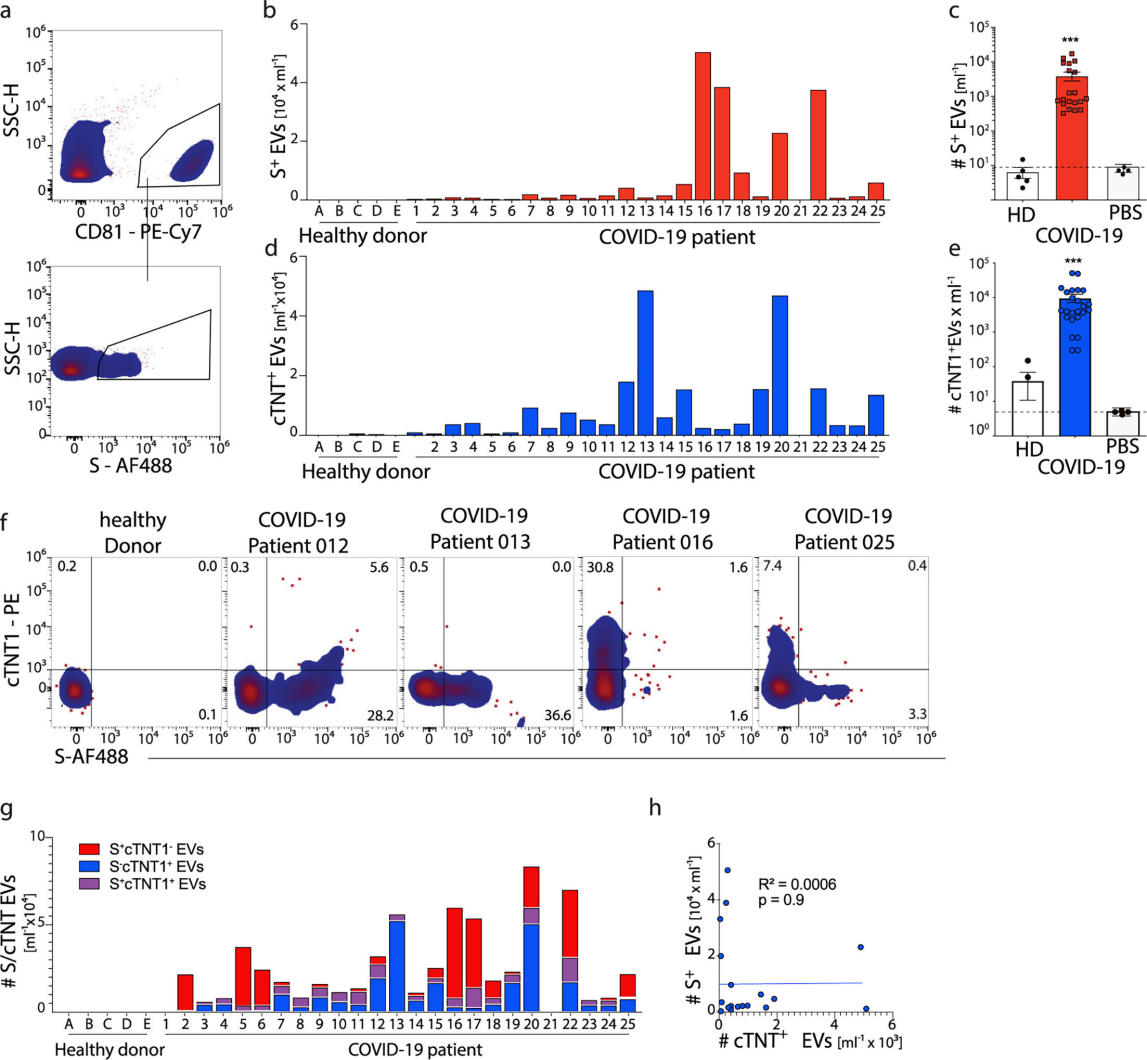
Identification of Spike-S^+^ and cTNT1^+^ EVs in the plasma of COVID-19 patients. (a–d) Plasma from COVID-19 patients or healthy individuals was stained with anti-CD81-PE-Cy7, anti-S-AF488 (clone 43A11) and anti-cTNT1-PE antibodies, followed by flow analysis. (a) Representative gating results. (b and d) Individual count per patient. (c and e) Cumulative count in combination with PBS-CTRL. (f and g) Representative and individual results for S and cTNT1. (h) Correlation of S^+^- and cTNT1^+^ EVs in COVID-19 patients. *n* (healthy) = 5; *n* (patients) = 25. Two-way ANOVA; ***P < 0.001, or simple linear regression was performed, as indicated.

By flow cytometric analysis of circulating EVs, we detected S protein expression on CD81^+^ EVs in plasma of 24 out of 25 COVID-19 patients, whereas no S-expressing CD81^+^ EVs were found in plasma from healthy individuals (Fig. 3a through c). In contrast to the analysis by flow cytometry, S-expressing EVs in plasma were not detected using ELISA or Western blot (Fig. S3b and d). There were substantial differences in the number of circulating S-expressing CD81^+^ EVs detected across COVID-19 patients investigated here (Fig. 3b and c). However, there was no difference in the overall number of CD81^+^ EVs found in the plasma of COVID-19 patients compared to healthy individuals (Fig. S3e). Furthermore, there was no correlation between the viral load detected in nasal swabs and the number of circulating S^+^ EVs (Fig. S3f through h).

We continued to determine the expression of cTNT1 on circulating CD81^+^ EVs and also found an increased number of circulating cTNT1^+^ EVs in the plasma of COVID-19 patients compared to healthy individuals (Fig. 3d and e). Notably, low numbers of cTNT1-expressing EVs were found in two healthy donors (Fig. 3e). To assess SARS-CoV-2 infection of cardiomyocytes in COVID-19 patients, we analyzed co-expression of S and cTNT1 on CD81^+^ EVs. This revealed a considerable heterogeneity among COVID-19 patients for the number of S^+^cTNT1^+^ EVs (Fig. 3f and g). Repeated measurements of EVs from four patients demonstrated the high reproducibility of the detection of S^+^ and cTnT1^+^ EVs by flow cytometry (Fig. S3i). There was no direct correlation between the number of circulating S^+^ and cTNT1^+^ EVs in COVID-19 patients (Fig. 3h). Altogether, these results indicated that the detection of circulating S^+^ and S^+^cTnT1^+^ EVs is possible in the plasma of COVID-19 patients and that it reflected SARS-CoV-2 infection of tissue cells and, in particular, cTnT1-expressing cardiomyocytes.

**Fig 4:**
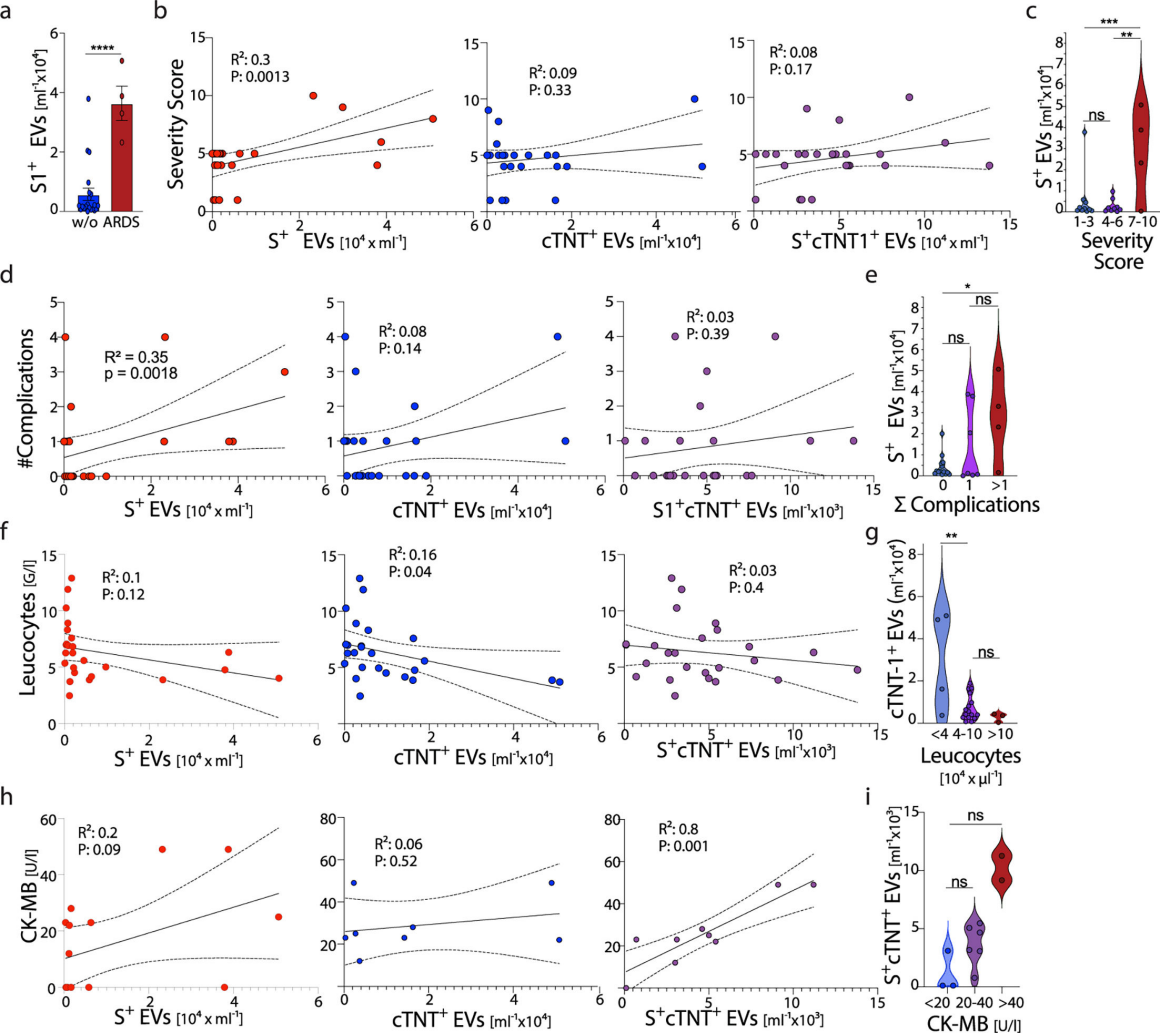
Correlation between EVs and clinically determined parameters. (a) The number of S^+^ EVs was plotted for patients with and without ARDS. (b, d, f, and h) correlation of S^+^ EVs (red) cTNT1^+^ EVs (blue) or S^+^cTNT1^+^ EVs (purple) with laboratory medical parameters; (b) the Lancet COVID-19 severity score (1), (d) sum of complications (ARDS, myocarditis, heart attack, acute kidney failure, pulmonary embolism, or peripheral thrombosis); (f) leukocyte count; and (h) CK-MB. (c, e, g, and i) Grouping of patients into the severity score (c), sum of complications (e), leukocyte count (g), and (i) CK-MB based on indicated EV numbers. Two-way ANOVA; ns *P* ≥ 0.05. *P < 0.05, **P < 0.01, ***P < 0.001, ****P < 0.00001 or simple linear regression was performed were indicated.

We next evaluated whether the detection of circulating S^+^ and cTnT1^+^ EVs at the time of admission might help to predict the disease course in COVID-19 patients over the next 2–3 weeks. Indeed, the number of circulating S^+^ EVs was higher in COVID-19 patients who later on progressed to develop an ARDS requiring mechanical ventilation (Fig. 4a). Moreover, the number of circulating S^+^ EVs but not cTNT1^+^ EVs or S^+^cTnT1^+^ EVs directly correlated with the COVID-19 severity score and the number of severe clinical complications developing over the following days, such as ARDS, myocarditis, heart attack, acute kidney failure, pulmonary embolism, and thrombotic events (Fig. 4b through e). However, we did not detect a direct correlation of circulating S^+^ EVs with routine laboratory parameters in COVID-19 patients at the time of analysis (Fig. S4) or with cytokines like IL-1, IL-6, IL-7, IL-8, IL-10, TNF, G-CSF, interferons type I and II or chemokines like CCL2, CCL5, and CXCL10 determined in plasma by bead array analysis (Fig. S5). For the number of circulating cTnT1-expressing EVs, however, we did not detect a correlation with the disease course in COVID-19 patients, routine laboratory parameters, and cytokines/chemokines (Fig. S4 and S5), but found an inverse correlation with the number of blood leukocytes (Fig. 4f and g). Of note, for the number of circulating S^+^cTnT1^+^ EVs, we detected a direct correlation with CK-MB levels (Fig. 4h and i). Thus, our analysis reveals that high numbers of circulating S^+^ EVs are not associated with direct organ damage but herald the development of clinical complications and severe disease courses.

## DISCUSSION

Circulating EVs reflect the state of the cells they are released from and bear the promise to serve as biomarkers for noninvasive diagnosis of viral infection or cancer, treatment monitoring, and disease prognosis (22, 23). UC and SEC for enrichment, followed by characterization of EVs by Western blot and bead-based immunoassays, are considered gold standards to characterize EVs (24–26). However, these methods bear limitations as they are not only cost-intensive, but due to their complexity, are prone to errors and investigate enriched EVs as a bulk rather than individual EVs (24–26). We have established a workflow for flow cytometry, which facilitates the precise multiparametric analysis of individual EVs derived from cell culture supernatant as well as patient plasma. Applied to a clinical context, our approach enabled the detection and characterization of EVs bearing markers of ongoing SARS-CoV-2 infection and derived from cardiomyocytes from the plasma of patients with COVID-19. This provides an advantage over previous reports (24–29) as it does not require initial enrichment by UC and enables the quantification of EVs according to their phenotypic characteristics, which allows for the analysis of small numbers of particular EVs in patients’ plasma.

COVID-19 pathogenesis remains insufficiently understood until today (30), but it is known that tissue and organ damage is inflicted by a dysregulated immune response (31, 32). In particular, SARS-CoV-2 infection of the cardiovascular system appears to contribute to a variety of complications such as myocarditis, vascular damage, arrhythmia, and thrombosis (12, 14, 33), and the degree of ongoing inflammation predicts severe disease courses (34, 35). Previous studies reported the presence of circulating EVs bearing SARS-CoV-2 proteins (22), such as S, that correlated directly with disease severity. Here, we address the question of whether the detection of SARS-CoV-2 S-bearing circulating EVs would allow for stratifying COVID-19 patients at the time of admission to the emergency department for the subsequent development of clinical complications and severe disease courses. There was no correlation between the number of circulating S-expressing EVs and any laboratory parameter indicating ongoing organ damage in the heart, liver, or kidney, and the plasma concentrations of immune mediators associated with a CRS, indicating that, at the time of admission, no grave dysregulation of immune responses was present causing tissue or organ damage. However, over the course of the next two weeks, we found that the abundance of circulating SARS-CoV-2 S-expressing EVs at the time of admission directly correlated with later development of inflammation, organ failure, and the COVID-19 severity score, which indicates that analysis of circulating EVs yields predictive information on the subsequent disease course of SARS-CoV-2 infected patients.

Notwithstanding this early prognostic information provided by quantifying the number of circulating S-expressing EVs, we did not detect a correlation between the number of cTNT1/S-expressing EVs and severe disease courses. Using iPS-derived human cardiomyocytes, we established that SARS-CoV-2 infection of these cells *in vitro* can be identified by analysis of their EVs bearing S on their surface. However, we failed to detect a correlation between cTNT1/S-expressing EVs and cardiovascular complications. This may be related to a sensitivity issue of the flow cytometry-based assay to detect very low frequencies of circulating cTNT1/S-expressing EVs, or may be the consequence of the absence of patients in our small study cohort who experienced SARS-CoV-2 infection of the heart. Studies involving a larger number of SARS-CoV-2-infected patients will be required to provide further insights into the question of whether cTNT1/S-expressing EVs predict the development of cardiovascular complications.

Thus, flow cytometry-based detection of circulating EVs and their analysis for evidence of S expression as a marker for ongoing SARS-CoV-2 infection proved to be useful at the time of admission of COVID-19 patients for the prediction of severe organ complications over the next two weeks. Beyond the PCR-based analysis of viral infection, the detection of viral replication in the organism through analysis of circulating viral antigen-expressing EVs may therefore provide important prognostic information in situations of a future viral pandemic.

## MATERIALS AND METHODS

### Generation of S-transduced HEK293T cells and supernatants

To generate EVs with high levels of SARS-CoV-2 spike S^FL^ (D614G), a constitutive S-expressing HEK293 cell line (CID4618, Helmholtz Zentrum München) was generated by transduction with a *S(B.1*) gene expression plasmid (#7413, Helmholtz Zentrum München). S-transduced and untransduced HEK293T cells were cultured in serum-free medium for 12 h, the cell culture supernatant was collected, centrifuged for 15 min at 2,000 × *g*, and for 10 min at 10,000 × *g* at 4°C to remove cells and cell debris, and stored at −80°C until further analysis.

### Anti-S antibody generation and production

Anti-S antibody was generated as reported. One antibody with high-affinity binding to S (43A11) was selected for further studies. Anti-S-antibodies were purified from hybridoma supernatant using a GammaBind Plus Sepharose (17088602, Cytiva) column, washed with PBS, and eluted in citric acid buffer at pH 2.7. Antibody preparations were further purified using a Superdex 200 prep grade (17104301, Cytiva) in PBS to obtain monomers and coupled to a fluorochrome (Alexa488) using the labeling kit A10235 (Thermo Fisher Scientific, Waltham, MA, USA) (17).

### Differentiation of human cardiomyocytes from iPS cells

The iPS cells were treated as previously described to induce the differentiation of functional cardiomyocytes (19). iPS-derived cardiomyocytes were cultured in DMEM containing 10% FCS (10 mM), HEPES (0.55 mM), arginine (0.272 mM), and asparagine. The cells were incubated in a humidified atmosphere with 5% CO_2_ at 37°C to reach confluence. Twelve hours before the supernatant was collected, the medium was removed, and the cells were washed with PBS. The cells were cultivated in a serum-free medium for 12 h. The cell culture supernatant was collected and centrifuged for 15 min at 2,000 × *g*, 20°C and for 10 min at 10,000 × *g* at 4°C to remove cells and cell debris. Samples were stored at −80°C until further analysis.

### Infection of iPSC-derived cardiomyocytes

iPSC-derived cardiomyocytes were infected with SARS-CoV-2 (B1) at an MOI of 1 in the EB2 medium. The SARS-CoV-2 isolate hCoV-19/Germany/BAV-PL-virotum-nacq/2020 (GISAID accession ID: EPI_ISL_582134) was derived from a nasopharyngeal swab, expanded *in vitro,* and aliquots were stored at −80°C until use. iPSC-derived cardiomyocytes were infected with SARS-CoV-2 at different multiplicities of infection. The cells were washed with PBS 2 h post-infection and further cultivated in EB2 medium for 22 h before analysis. The seasonal coronavirus hCoV-NL63 (AY567487) (36) was used as a control to infect iPS-derived cardiomyocytes.

### Plasma samples from healthy individuals and from COVID-19 patients

A clinical study (approval 471/20 S from the ethics committee of the Technical University of Munich) was performed at the TUM University Hospital between February and June 2021 to investigate circulating EVs in blood samples from COVID-19 patients. Evidence of SARS-CoV-2 infection was obtained by a positive RT‒PCR result. Blood samples from patients were obtained at the time of admission to the emergency department of the TUM University Hospital. Human whole blood was drawn using butterfly syringes and EDTA monovettes and transferred to 15 mL reaction tubes and centrifuged for 15 min at 2,000 × *g* at 20°C. Platelet-free plasma (PPP) was collected and transferred to 2 mL reaction tubes and centrifuged for 15 min at 10,000 × *g* at 20°C to remove remaining cells and cellular debris. PFP was collected and transferred into fresh reaction tubes. The samples were aliquoted into fresh reaction tubes and stored at −80°C until further use.

### Flow cytometric analysis of EVs

Flow cytometry analyses were performed using a Sony Spectral Flow Cytometer SA3800 equipped with two lasers (405 and 488 nm), a Sony Spectral Cytometer ID7000 with five lasers (355, 405, 488, 561, and 638 nm) (both from Sony Biotechnology, Japan) or a Beckman Coulter CytoFLEX LX Flow cytometer equipped with four lasers (405, 488, 561, and 638 nm) (BA16019, Beckman Coulter, USA).

The system settings listed in Table 1 were used.

**TABLE 1:** Instrument settings for flow cytometer

	Cytoflex LX	SA3800	ID7000
Flow rate	Slow	1	1
Threshold	V-SSC-H 2000 High	SSC 0.1%	SSC 4.5%
Gain			
FSC	89	11%	17
SSC	58	28%	6.25
V-SSC	300	n/a^*a*^	n/a
Fluorescence PMT voltage	1,200	65%	5.66
Compensation	None	WLSM	WLSM
Additional settings	None	No event check	No event check

^*a*^ n/a, not applicable.

Samples were measured in plate-loader mode with 1,000 events per second for 10 min. Flow cytometry data were analyzed with FlowJo Software 10.2 (TreeStar Inc., Ashland, OR, USA).

### Immunophenotyping of EVs from plasma or cell culture supernatant EVs

A total of 10 µL of plasma or cell culture supernatant was incubated with the following antibodies: anti-CD63 (1 ng/µL, Clone H5C6), anti-CD81 (1 ng/µL, Clone 5A6), anti-CD9 (1 ng/µL, Clone HI9a) (BioLegend, San Diego, CA, USA), anti-HLA-ABC (1 ng/µL Clone W6/32) (Sony Biotechnology, San Jose, CA, USA), anti-S (43A11; Helmholtz), and anti-TroponinT (1 ng/µL, Clone REA400) (Miltenyi Biotech, Bergisch Gladbach, Germany) and incubated for 30 min at 20°C in the dark. The samples were diluted with PBS (Gibco Life Technologies, Thermo Fisher Scientific). As a negative control for antibody background, 10 µL of PBS was stained with the appropriate antibody and diluted analogously to plasma or cell culture supernatant. Cell counting beads (Thermo Fisher Scientific) were included in each sample prior to flow cytometry to allow for absolute cell quantification.

### Cryo-electron microscopy (cryo-EM)

The samples were processed for cryo-EM as follows. A 4 µL aliquot was deposited on an EM grid coated with a perforated carbon film; the liquid was blotted from the backside of the grid, and the grid was immersed in liquid ethane using a Leica EMCPC cryo-chamber. The EM grids were stored under liquid nitrogen prior to EM observation. Cryo-EM was performed with a Tecnai F20 (FEI, USA) microscope equipped with a USC1000-SSCCD camera (Gatan, USA).

### Western blot

Concentrated S^+^ EVs or control EVs (S^−^) were lysed in nonreducing 5× Laemmli buffer and separated by SDS‒PAGE. For western blot analysis of Spike-S in plasma from COVID-19 patients or healthy donors, 2–12 µL of plasma was diluted with 5× Laemmli buffer and ddH_2_O to a final volume of 15 µL and separated by SDS‒PAGE. Proteins were transferred to nitrocellulose membranes (GE Healthcare Life Sciences) by semidry blotting (Bio-Rad), and the membranes were blocked for 1 h in 5% (wt/vol) nonfat milk in ddH_2_O at RT. The membranes were incubated overnight at 7°C with primary anti-Spike-S antibody (43A11, rat IgG, Helmholtz Zentrum München) at a 1:2,000 dilution in 5% (wt/vol) nonfat milk (Roth) in ddH_2_O, washed three times in TBST (Tris-buffered saline with 0.1% Tween-20) and incubated for 1 at RT with horseradish peroxidase (HRP)-conjugated anti-rat antibody (1112-035-062, goat anti-rat IgG, Jackson ImmunoResearch Europe) at a 1:20,000 dilution in 5% (wt/vol) nonfat milk in PBST (phosphate-buffered saline with 0.1% Tween-20). After three washes in TBST, the blots were incubated with enhanced chemiluminescence reagent (GE Healthcare) and imaged using a Fusion FX (Vilber).

### Enzyme-linked immunosorbent assay (ELISA)

For the quantification of the spike protein in samples from various sources, a sandwich ELISA was developed using two anti-spike antibodies with orthogonal epitopes. The wells of a Nunc MaxiSorp plate (Thermo Fisher Scientific) were coated for 5 h at 20°C with 2 µg mL^−1^ anti-Spike-S capture antibody (55E10, rat IgG, Helmholtz Zentrum München) or isotype control in PBS. After washing with PBST (PBS + 0.05% Tween-20), free binding sites were blocked for 2 h at 20°C in 5% (wt/vol) nonfat milk (Roth) in PBS. Samples of recombinant Spike protein (S1 + S2 extracellular domains, 40589-V08B1, Sino Biological), *in vitro*-derived S^+^ EVs, or plasma from COVID-19 patients (Technical University of Munich) were diluted as indicated in PBS containing 10 µg mL^−1^ isotype antibody (Helmholtz Zentrum München) to eliminate nonspecific plasma-derived background (37) before being incubated for 16 h at 7°C in a coated plate. After washing, HRP-conjugated anti-Spike-S detection antibody (43A11, rat IgG, Helmholtz Zentrum München) was added for 2 h at 20°C at a 1:500 dilution in 5% (wt/vol) nonfat milk in PBS, after which the samples were rewashed and incubated at 20°C with 100 µL of TMB substrate reagent (BD555214, Becton Dickinson). The reaction was stopped by adding 50 µL of 1 M H_2_SO_4,_ and the absorbance was measured at 450 nm in a CLARIOstar Plus (BMG Labtech). Data analysis was performed with GraphPad Prism.

### RT-qPCR for the detection of viral infection

RNA extraction was performed using RNeasy FFPE Kit (QIAGEN). Fold gene expression was determined using the 2^–∆∆Ct^ – Method (non-detected targets were assigned a CT of 40, and averaged gene expression in uninfected cells was used as the reference) using Quantstudio 5, Applied Biosystems.

Primer : SARS-CoV-2 N: fw 5′-TTACAAACATTGGCCGCAAA-3′; rev 5′-GCGCGACATTCCGAAGAA-3′.

NL-63 RF2: fw 5′-CTTCTGGTGACGCTAGTACAGCTTAT-3′; rev 5′-AGACGTCGTTGTAGATCCCTAACAT-3′; Housekeeping: RPLP0: 5′-GATAACCAGTCGAAGTCACCTAGTTC-3′; 5′ GATAACCAGTCGAAGTCACCTAGTTC-3′).

### NTA measurement of EVs

NTA was performed with a ZetaView PMX110 instrument (ParticleMetrix), and the corresponding software (ZetaView 8.04.02) was used to measure the number and size distribution of the EVs. The samples were diluted in filtered PBS to achieve a vesicle concentration of approximately 1 × 10^7^ mL^−1^. The pre-acquisition parameters were set to a sensitivity of 75, a shutter speed of 50, a frame rate of 30 frames per second, and a trace length of 15. The post-acquisition parameters were set to a minimum brightness of 20, a minimum size of 5 pixels, and a maximum size of 1,000 pixels.

### Statistical analysis

Statistical analyses were performed with GraphPad Prism 6 (GraphPad Software). Differences between groups were calculated using the Student’s two-way unpaired *t*-test, two-way ANOVA, the Mantel‒Cox test, or simple linear regression. Statistical significance is depicted as the *P* value (*P < 0.05; **P < 0.01; ***P < 0.001; and ****P < 0.00001).

## Data Availability

All clinical data are summarized in Table S1. Additional data are only available on request due to ethical considerations.

## SUPPLEMENTAL MATERIAL

Figure S1 jvi.01189-25-s0001.eps Detection of S EVs in cell culture supernatants.

Figure S2 jvi.01189-25-s0002.eps Infection of cardiomyocytes.

Figure S3 jvi.01189-25-s0003.eps Identification of Spike-S^+^ and cTNT1^+^ EVs in the plasma of COVID-19 patients.

Figure S4 jvi.01189-25-s0004.eps Correlation of S^+^ or cTNT1 EVs with clinical laboratory parameters.

Figure S5 jvi.01189-25-s0005.eps Correlation of S^+^ or cTNT1^+^ EVs with routine laboratory parameters.

Supplemental legends jvi.01189-25-s0006.docx Legends for supplemental material.

Graphical abstract jvi.01189-25-s0007.eps Infection with SARS-CoV-2 is diagnosed by detecting viral RNA by PCR obtained from nasopharyngeal swabs. While this approach provides essential diagnostic information on the infectious agent, it does not provide information on the infection of organs that may be important for predicting the disease course. Here we demonstrate that SARS-CoV-2 proteins and heart-muscle-specific molecules are detected on circulating extracellular vesicles and that the detection of SARS-CoV-2 protein-expressing EVs predicted a severe disease course in COVID-19 patients.

Table S1 jvi.01189-25-s0008.xlsx Patient information and clinical chemistry.

ASM does not own the copyrights to Supplemental Material that may be linked to, or accessed through, an article. The authors have granted ASM a non-exclusive, world-wide license to publish the Supplemental Material files. Please contact the corresponding author directly for reuse.

## References

[1] Wu F, Zhao S, Yu B, Chen Y-M, Wang W, Song Z-G, Hu Y, Tao Z-W, Tian J-H, Pei Y-Y, Yuan M-L, Zhang Y-L, Dai F-H, Liu Y, Wang Q-M, Zheng J-J, Xu L, Holmes EC, Zhang Y-Z. 2020. A new coronavirus associated with human respiratory disease in China. Nature 579:265–269. doi:10.1038/s41586-020-2008-3 32015508 PMC7094943

[2] Zhou P, Yang X-L, Wang X-G, Hu B, Zhang L, Zhang W, Si H-R, Zhu Y, Li B, Huang C-L, et al.. 2020. A pneumonia outbreak associated with a new coronavirus of probable bat origin. Nature 579:270–273. doi:10.1038/s41586-020-2012-7 32015507 PMC7095418

[3] Bikdeli B, Madhavan MV, Jimenez D, Chuich T, Dreyfus I, Driggin E, Nigoghossian CD, Ageno W, Madjid M, Guo Y, et al.. 2020. COVID-19 and thrombotic or thromboembolic disease: implications for prevention, antithrombotic therapy, and follow-up: JACC state-of-the-art review. J Am Coll Cardiol 75:2950–2973. doi:10.1016/j.jacc.2020.04.031 32311448 PMC7164881

[4] Msemburi W, Karlinsky A, Knutson V, Aleshin-Guendel S, Chatterji S, Wakefield J. 2023. The WHO estimates of excess mortality associated with the COVID-19 pandemic. Nature 613:130–137. doi:10.1038/s41586-022-05522-2 36517599 PMC9812776

[5] Lamers MM, Haagmans BL. 2022. SARS-CoV-2 pathogenesis. Nat Rev Microbiol 20:270–284. doi:10.1038/s41579-022-00713-0 35354968

[6] Hoffmann M, Kleine-Weber H, Schroeder S, Krüger N, Herrler T, Erichsen S, Schiergens TS, Herrler G, Wu N-H, Nitsche A, Müller MA, Drosten C, Pöhlmann S. 2020. SARS-CoV-2 cell entry depends on ACE2 and TMPRSS2 and is blocked by a clinically proven protease inhibitor. Cell 181:271–280. doi:10.1016/j.cell.2020.02.052 32142651 PMC7102627

[7] Wong DWL, Klinkhammer BM, Djudjaj S, Villwock S, Timm MC, Buhl EM, Wucherpfennig S, Cacchi C, Braunschweig T, Knüchel-Clarke R, Jonigk D, Werlein C, Bülow RD, Dahl E, von Stillfried S, Boor P. 2021. Multisystemic cellular tropism of SARS-CoV-2 in autopsies of COVID-19 patients. Cells 10:1900. doi:10.3390/cells10081900 34440669 PMC8394956

[8] Machkovech HM, Hahn AM, Garonzik Wang J, Grubaugh ND, Halfmann PJ, Johnson MC, Lemieux JE, O’Connor DH, Piantadosi A, Wei W, Friedrich TC. 2024. Persistent SARS-CoV-2 infection: significance and implications. Lancet Infect Dis 24:e453–e462. doi:10.1016/S1473-3099(23)00815-0 38340735

[9] Blagoeva V, Hodzhev V, Uchikov P, Dobreva-Yatseva B, Stoyanova R, Shterev M, Atiq S, Prasad A, Shankar Babu S. 2025. Clinical course and mortality predictors in adult hospitalized patients with COVID-19 infection-a retrospective cohort study. Medicina (Kaunas) 61:579. doi:10.3390/medicina61040579 40282870 PMC12028986

[10] Huang C, Wang Y, Li X, Ren L, Zhao J, Hu Y, Zhang L, Fan G, Xu J, Gu X, et al.. 2020. Clinical features of patients infected with 2019 novel coronavirus in Wuhan, China. Lancet 395:497–506. doi:10.1016/S0140-6736(20)30183-5 31986264 PMC7159299

[11] Driggin E, Madhavan MV, Bikdeli B, Chuich T, Laracy J, Biondi-Zoccai G, Brown TS, Der Nigoghossian C, Zidar DA, Haythe J, Brodie D, Beckman JA, Kirtane AJ, Stone GW, Krumholz HM, Parikh SA. 2020. Cardiovascular considerations for patients, health care workers, and health systems during the COVID-19 pandemic. J Am Coll Cardiol 75:2352–2371. doi:10.1016/j.jacc.2020.03.031 32201335 PMC7198856

[12] Shi S, Qin M, Shen B, Cai Y, Liu T, Yang F, Gong W, Liu X, Liang J, Zhao Q, Huang H, Yang B, Huang C. 2020. Association of cardiac injury with mortality in hospitalized patients with COVID-19 in Wuhan, China. JAMA Cardiol 5:802–810. doi:10.1001/jamacardio.2020.0950 32211816 PMC7097841

[13] Dmytrenko O, Das S, Kovacs A, Cicka M, Liu M, Scheaffer SM, Bredemeyer A, Mack M, Diamond MS, Lavine KJ. 2024. Infiltrating monocytes drive cardiac dysfunction in a cardiomyocyte-restricted mouse model of SARS-CoV-2 infection. J Virol 98:e0117924. doi:10.1128/jvi.01179-24 39207134 PMC11406924

[14] Guo T, Fan Y, Chen M, Wu X, Zhang L, He T, Wang H, Wan J, Wang X, Lu Z. 2020. Cardiovascular implications of fatal outcomes of patients with coronavirus disease 2019 (COVID-19). JAMA Cardiol 5:811–818. doi:10.1001/jamacardio.2020.1017 32219356 PMC7101506

[15] Buzas EI. 2023. The roles of extracellular vesicles in the immune system. Nat Rev Immunol 23:236–250. doi:10.1038/s41577-022-00763-8 35927511 PMC9361922

[16] Iannotta D, A A, Kijas AW, Rowan AE, Wolfram J. 2024. Entry and exit of extracellular vesicles to and from the blood circulation. Nat Nanotechnol 19:13–20. doi:10.1038/s41565-023-01522-z 38110531 PMC10872389

[17] Roessler J, Pich D, Krähling V, Becker S, Keppler OT, Zeidler R, Hammerschmidt W. 2023. SARS-CoV-2 and Epstein-Barr virus-like particles associate and fuse with extracellular vesicles in virus neutralization tests. Biomedicines 11:2892. doi:10.3390/biomedicines11112892 38001893 PMC10669694

[18] Roessler J, Pich D, Albanese M, Wratil PR, Krähling V, Hellmuth JC, Scherer C, von Bergwelt-Baildon M, Becker S, Keppler OT, Brisson A, Zeidler R, Hammerschmidt W. 2022. Quantitation of SARS-CoV-2 neutralizing antibodies with a virus-free, authentic test. PNAS Nexus 1:pgac045. doi:10.1093/pnasnexus/pgac045 36382127 PMC9645495

[19] Moretti A, Laugwitz KL, Dorn T, Sinnecker D, Mummery C. 2013. Pluripotent stem cell models of human heart disease. Cold Spring Harb Perspect Med 3:a014027. doi:10.1101/cshperspect.a014027 24186488 PMC3808770

[20] Moretti A, Bellin M, Jung CB, Thies T-M, Takashima Y, Bernshausen A, Schiemann M, Fischer S, Moosmang S, Smith AG, Lam JT, Laugwitz K-L. 2010. Mouse and human induced pluripotent stem cells as a source for multipotent Isl1^+^ cardiovascular progenitors. FASEB J 24:700–711. doi:10.1096/fj.09-139477 19850773

[21] Klemm T, Ebert G, Calleja DJ, Allison CC, Richardson LW, Bernardini JP, Lu BG, Kuchel NW, Grohmann C, Shibata Y, et al.. 2020. Mechanism and inhibition of the papain-like protease, PLpro, of SARS-CoV-2. EMBO J 39:e106275. doi:10.15252/embj.2020106275 32845033 PMC7461020

[22] Aharon A, Dangot A, Kinaani F, Zavaro M, Bannon L, Bar-Lev T, Keren-Politansky A, Avivi I, Jacob G. 2023. Extracellular vesicles of COVID-19 patients reflect inflammation, thrombogenicity, and disease severity. Int J Mol Sci 24:5918. doi:10.3390/ijms24065918 36982991 PMC10054500

[23] Alberro A, Iparraguirre L, Fernandes A, Otaegui D. 2021. Extracellular vesicles in blood: sources, effects, and applications. Int J Mol Sci 22:8163. doi:10.3390/ijms22158163 34360924 PMC8347110

[24] Bano R, Ahmad F, Mohsin M. 2021. A perspective on the isolation and characterization of extracellular vesicles from different biofluids. RSC Adv 11:19598–19615. doi:10.1039/d1ra01576a 35479207 PMC9033677

[25] Chen J, Li P, Zhang T, Xu Z, Huang X, Wang R, Du L. 2022. Review on strategies and technologies for exosome isolation and purification. Front Bioeng Biotechnol 9. doi:10.3389/fbioe.2021.811971 PMC8766409 35071216

[26] Gorgzadeh A, Nazari A, Ali Ehsan Ismaeel A, Safarzadeh D, Hassan JAK, Mohammadzadehsaliani S, Kheradjoo H, Yasamineh P, Yasamineh S. 2024. A state-of-the-art review of the recent advances in exosome isolation and detection methods in viral infection. Virol J 21:34. doi:10.1186/s12985-024-02301-5 38291452 PMC10829349

[27] Théry C, Amigorena S, Raposo G, Clayton A. 2006. Isolation and characterization of exosomes from cell culture supernatants and biological fluids. Curr Protoc Cell Biol Chapter 3:Unit doi:10.1002/0471143030.cb0322s30 18228490

[28] Cocucci E, Meldolesi J. 2015. Ectosomes and exosomes: shedding the confusion between extracellular vesicles. Trends Cell Biol 25:364–372. doi:10.1016/j.tcb.2015.01.004 25683921

[29] Wang J-M, Li Y-J, Wu J-Y, Cai J-X, Wen J, Xiang D-X, Hu X-B, Li W-Q. 2021. Comparative evaluation of methods for isolating small extracellular vesicles derived from pancreatic cancer cells. Cell Biosci 11:37. doi:10.1186/s13578-021-00550-3 33568197 PMC7877077

[30] Yang OO. 2025. The immunopathogenesis of SARS-CoV-2 infection: overview of lessons learned in the first 5 years. J Immunol 214:1095–1104. doi:10.1093/jimmun/vkaf033 40180332

[31] Moss P. 2022. The T cell immune response against SARS-CoV-2. Nat Immunol 23:186–193. doi:10.1038/s41590-021-01122-w 35105982

[32] Mohandas S, Jagannathan P, Henrich TJ, Sherif ZA, Bime C, Quinlan E, Portman MA, Gennaro M, Rehman J, RECOVER Mechanistic Pathways Task Force. 2023. Immune mechanisms underlying COVID-19 pathology and post-acute sequelae of SARS-CoV-2 infection (PASC). Elife 12:e86014. doi:10.7554/eLife.86014 37233729 PMC10219649

[33] Dherange P, Lang J, Qian P, Oberfeld B, Sauer WH, Koplan B, Tedrow U. 2020. Arrhythmias and COVID-19: a review. JACC Clin Electrophysiol 6:1193–1204. doi:10.1016/j.jacep.2020.08.002 32972561 PMC7417167

[34] Sharifpour M, Rangaraju S, Liu M, Alabyad D, Nahab FB, Creel-Bulos CM, Jabaley CS, Emory COVID-19 Quality & Clinical Research Collaborative. 2020. C-Reactive protein as a prognostic indicator in hospitalized patients with COVID-19. PLoS One 15:e0242400. doi:10.1371/journal.pone.0242400 33216774 PMC7679150

[35] Luan YY, Yin CH, Yao YM. 2021. Update advances on C-reactive protein in COVID-19 and other viral infections. Front Immunol 12:720363. doi:10.3389/fimmu.2021.720363 34447386 PMC8382792

[36] van der Hoek L, Pyrc K, Jebbink MF, Vermeulen-Oost W, Berkhout RJM, Wolthers KC, Wertheim-van Dillen PME, Kaandorp J, Spaargaren J, Berkhout B. 2004. Identification of a new human coronavirus. Nat Med 10:368–373. doi:10.1038/nm1024 15034574 PMC7095789

[37] Degn SE, Andersen SH, Jensen L, Thiel S, Jensenius JC. 2011. Assay interference caused by antibodies reacting with rat kappa light-chain in human sera. J Immunol Methods 372:204–208. doi:10.1016/j.jim.2011.06.030 21771595

