# Supplementary material for: Prediction of COVID-19 disease progression by multiparametric analysis of circulating extracellular vesicles with flow cytometry
Source: J Virol. 2025 Sep 23;99(10):e01189-25. doi: 10.1128/jvi.01189-25 (PMC12548395; doi:10.1128/jvi.01189-25)
Supplement: Supplemental legends — Legends for supplemental material. [file jvi.01189-25-s0006.docx]

***Supplementary Figure 1: Detection of S EVs in cell culture supernatants***

*SARS-CoV-2-S-transduced HEK293T cells were cultured in serum-free media for 12 hours. Cell culture supernatants were centrifuged (10,000 × g) to remove the cells and cell fragments before further analysis.* ***a****, Quantification EVs in cell culture supernatants by nanoparticle tracking analysis (NTA).* ***b,*** *EVs from HEK293T-S^+^ and -S^-^ cells were enriched by ultracentrifugation (UC). Western blot of EV- and cell- lysates are shown.* ***c****,* *A direct comparison was made between different purification techniques. The cell culture medium of HEK293T-S^+^ cells was analysed directly, enriched by UC and resuspended in 10% volume, purified by size exclusion chromatography (SEC) or isolated by the combination of SEC and UC, and also analysed by flow cytometry. The results are plotted as a representative dotplot (****c****) or the quantification (****d****) of three independent experiments.*

***Supplementary Figure 2: Infection of cardiomyocytes****.* ***a****, Cardiomyocytes were infected with SARS-CoV2-GFP (a) or SARS-CoV2 or hCoV-NL63 (MOI: 0.1) (b). After 24 hours, cells were analysed for the expression of GFP using the Incucyte system (a) or by RT-qPCR (b). Relative expression of SARS-CoV-2 nucleocapsid and HCoV-NL63 release factor 2 in cardiomyocytes derived from iPSCs following infection with the respective virus at a MOI of 0.1 relative to RPLP0. 3 biological replicates per condition. Each data point is the mean of 3 technical PCR replicates. Mock = Uninfected.* ***c****, Infection of cardiomyocytes with SARS-CoV-2. A confluent layer of iPSC-induced, beating cardiomyocytes that were either uninfected, infected with SARS-CoV-2 (B1.1.7) or with hCoV-NL63 (MOI of 1). The cells were washed after 2 h, and the supernatant was collected after 12 h, followed by staining with anti-CD81, anti-cTNT1 and anti-Spike-1 antibodies. The supernatant was stained with anti-Spike-1 and anti-cTNT1 pregating on anti-CD81. Representative results from n = 3 are shown.*

***Supplementary Figure 3:*** ***Identification of Spike-S^+^ and cTNT1^+^ EVs in the plasma of COVID-19 patients. a****, EV analysis of plasma from healthy donors. Ten microliters of plasma were stained with 1 ng/µl of anti-CD9-PerCP-Cy5.5, anti-CD63-BV711, anti-CD81-PE-Cy7, or anti-HLA-ABC-PE-Cy5, incubated for 30 min at RT, diluted 1/1000 with PBS, and analysed by flow cytometry. The number of EVs was determined by counting the number of beads. Representative dot plots (n = 3) are shown****. b, c****, Standard curve and ELISA detection of Spike-S in plasma.* ***d****, Western Blot analysis of plasma for S* ***e****, Quantification of CD81^+^ EVs in plasma from COVID-19 patients and healthy individuals.* ***f- h,*** *correlation of the viral load with S^+^ EVs, cTNT1^+^ EVs or S^+^cTNT1^+^ EVs.* ***i****, Repeated analysis of S^+^ and cTNT1^+^ EVs was conducted in the serum of patients. The flow-based analysis was performed in three independent experiments, and the number of EVs was plotted* *with the coefficient of variation (CV) in %.*

***Supplementary Figure 4: Correlation of S^+^ or cTNT1 EVs with clinical laboratory parameters****. The clinical chemistry parameters creatinine, bilirubin, g-GT, hs-troponin, creatine kinase, CK-MB, CRP, LDH, procalcitonin, leucocytes, D-dimer, and thrombocytes were correlated with S^+^ EVs (red), cTNT1^+^ EVs (blue) or S^+^cTNT1^+^ EVs (purple). Simple linear regression was performed, and the R² and p-value were plotted.*

***Supplementary Figure 5: Correlation of S^+^ or cTNT1^+^ EVs with routine laboratory parameters****.* ***a****, The* cytokines/chemokines *cytokines IL-1a, IL-2, IL-6, IL-7, IL-8, IL-10, CCL2, CCL5, CXM10, G-CSF, IFN-α, IFN-γ and TNF* were correlated with S^+^ EVs (red), cTNT1^+^ EVs (blue) or S^+^cTNT1^+^ EVs (purple). Simple linear regression was performed, and the R² and p-value were plotted.

***Supplementary Table 1: Patient information and clinical chemistry***

*Age, sex, Severity score, symptoms, existing comorbidities, laboratory values, therapy and medication, complications, SARS-CoV-2 variant and viral load was listed for the individual patients.*
